# Carcinoma ex pleomorphic adenoma of soft palate with cavernous sinus invasion

**DOI:** 10.1186/1477-7819-8-24

**Published:** 2010-03-30

**Authors:** Hsuan-Ho Chen, Li-Yu Lee, Shy-Chyi Chin, I-How Chen, Chun-Ta Liao, Shiang-Fu Huang

**Affiliations:** 1 Department of Otolaryngology, Chang Gung Memorial Hospital and Chang Gung University, Taiwan; 2 Department of Pathology, Chang Gung Memorial Hospital and Chang Gung University, Taiwan; 3 Department of Radiology, Chang Gung Memorial Hospital and Chang Gung University, Taoyuan, Taiwan

## Abstract

**Background:**

Carcinoma ex pleomorphic adenoma (CXPA) is an aggressive salivary gland malignancy and rare in minor salivary gland. A soft palate CXPA initially presenting as direct cavernous sinus (CS) invasion is very rare.

**Case Presentation:**

A 60-year-old male had a 3-month history of a small soft palatal mass with progressing left cheek numbness, proptosis, and disturbed vision. Biopsy of soft palatal tumor showed pleomorphic adenoma. Magnetic resonance imaging showed a tumor involving left maxilla, and extended from pterygopalatine fossa, inferior orbital fissure to CS. Excision of tumor revealed CXPA. Adjuvant concomitant chemo-radiation therapy (CCRT) was given. The tumor recurred 5 months later in left CS which was re-treated with CCRT. The disease status was stable at 2 years after the diagnosis of CXPA.

**Conclusion:**

We present this case to emphasize that patients with symptoms such as facial numbness, proptosis and disturbed vision should be carefully investigated for lesions invading CS by perineural spread.

## Background

Carcinoma ex-pleomorphic adenoma (CXPA) is a rare, aggressive, poorly understood malignancy that usually develops in a primary or recurrent pleomorphic adenoma (PA) [1]. The pathogenesis has not been well understood. CXPA accounts for 3.6% of all salivary neoplasms and for 11.7% of salivary malignancies [2]. Less than 7% of the cases occur in the palatal minor salivary glands [3]. Misdiagnosis is not uncommon because the carcinoma may represent various subtypes in the residual PA on microscopic examination [1]. Imaging study of CXPA usually presents as non-specific characteristics [4]. We describe a 60-year-old male diagnosed as a CXPA of the soft palate with direct cavernous sinus (CS) invasion. The initial presentations of his disease were left cheek numbness, proptosis and blurred vision which were rarely reported in the literature [5].

## Case presentation

**Figure 1:**
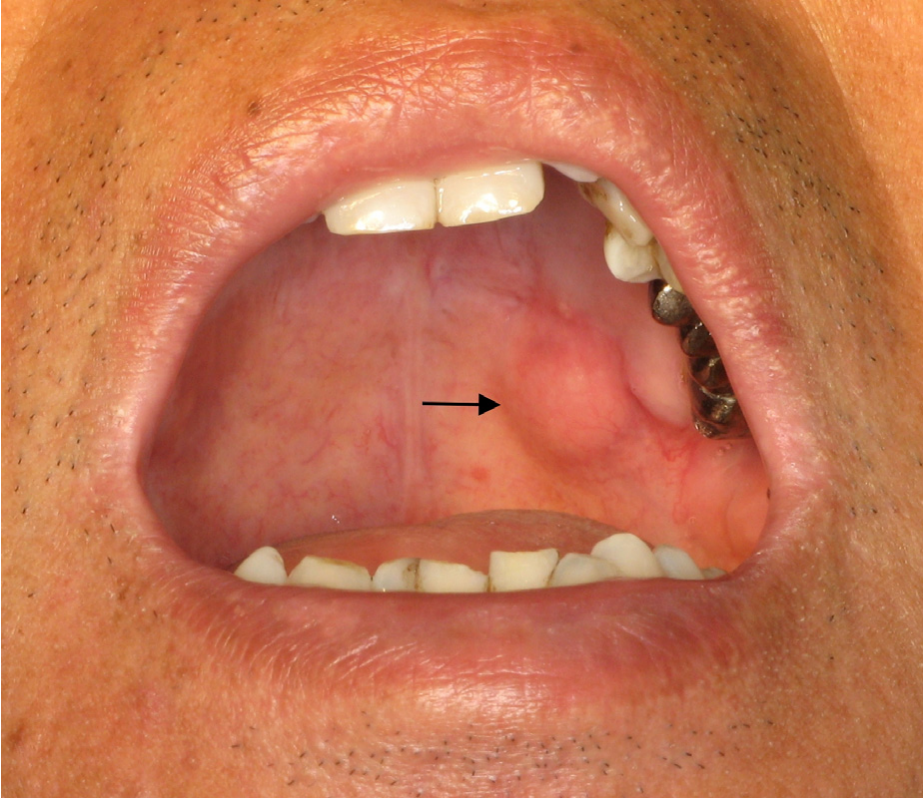
**A tumor measuring 1.2 × 1.0 cm located in left soft palate (arrow)**.

A 60-year-old male visited our hospital for left cheek numbness for 3 months. A painless soft palatal mass was noticed and became more prominent recently (Figure 1). Physical examinations of the head and neck revealed an area of numbness in the distribution of 2^nd ^division of trigeminal nerve, proptosis, and disturbed visual acuity at the level of counting fingers. He also had left esotropia which was due to traumatic left abducens nerve palsy by saw dust 10 months ago. Biopsy of the tumor revealed PA.

**Figure 2:**
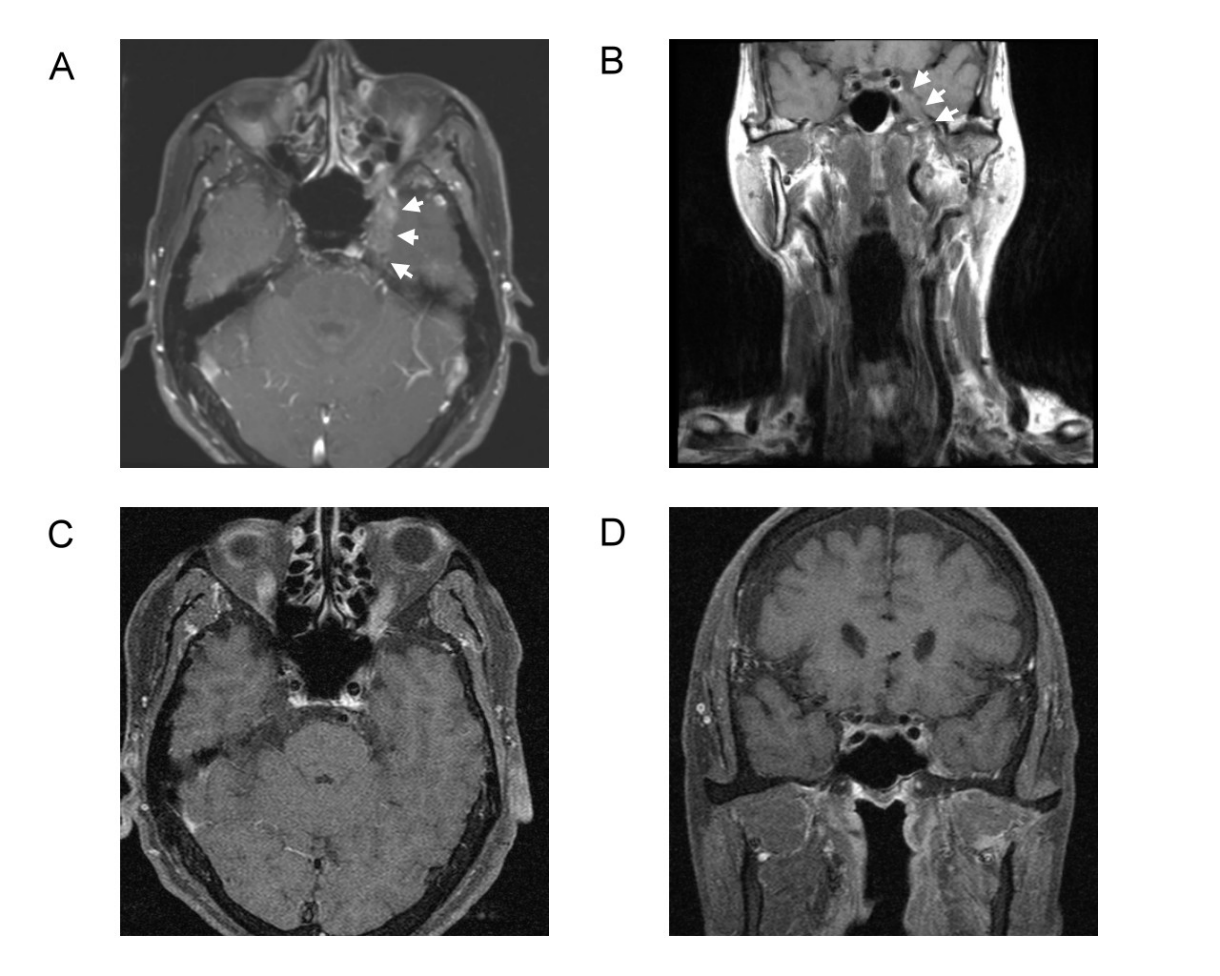
**T1-weighted magnetic resonance imaging scans** (A) Axial section shows irregular soft tissue mass in left cavernous sinus (arrows) before treatment, (B) Coronal section shows swelling of left parapharynx and masticator space and tumor invading into left cavernous sinus (arrows) before treatment, (C) Axial section shows remission of tumor 3 months after CCRT, (D) Coronal section shows remission of tumor 3 months after CCRT.

**Figure 3:**
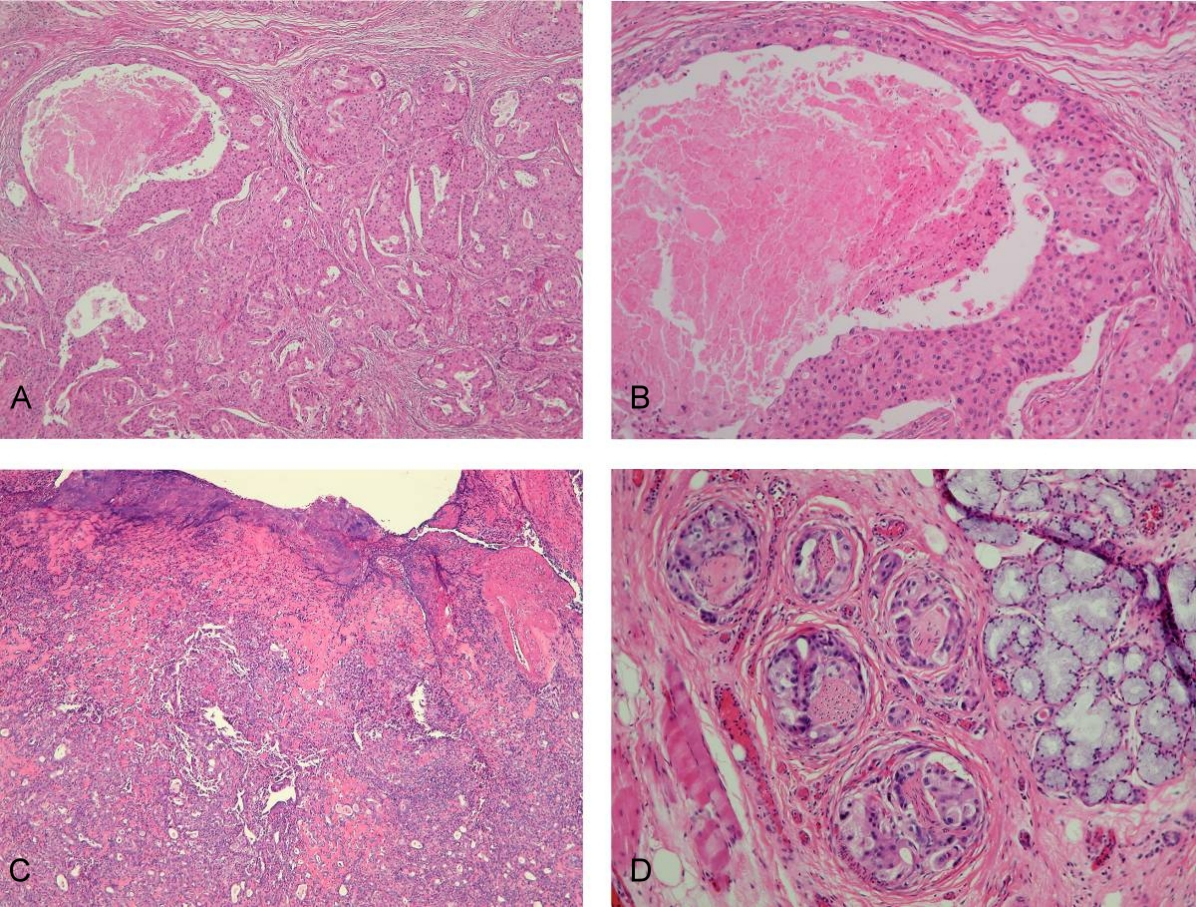
**Microscopic findings**. (A) Pleomorphic adenoma in a chondromyxoid, fibrotic and sclerotic stroma with the malignant areas of carcinoma component consisting epithelial clusters with necrosis. (H&E, 40×), (B) Carcinoma component is composed of cells with pleomorphic nucleus, mitotic activity and comedo-like necrosis. (H&E, 100×), (C) The carcinoma ex pleomorphic adenoma consisting benign pleomorphic adenoma and poorly differentiated carcinoma with the ulcerative epithelial surface. (H&E, 40×), (D) Tumor cells presented the perineural invasion (H&E, 400×).

Computed tomography of head and neck showed asymmetry with some bulging contour at palatal region. Magnetic resonance imaging (MRI) arranged revealed irregular soft tissue mass involving left maxilla, and extended from pterygopalatine fossa, inferior orbital fissure to CS with bony destruction of the skull base (Figure 2). We performed total maxillectomy with free flap reconstruction intending to radically excise the tumor. At surgery, we explored the pterygopalatine fossa but complete excision of tumor was infeasible at the skull base. After excision of the tumor, immunohistochemical study revealed actin (+), CD10(+), Calponin (+), and CK14(+). Final pathologic report was CXPA of salivary duct carcinoma subtype, with perineural, lymphatic and bony invasion (Figure 3). Concurrent chemo-radiotherapy (CCRT) was given after surgery due to positive resection margin and advanced tumor stage. The tumor status was completely remitted without deterioration of visual acuity, proptosis and slightly improvement of left check numbness (Figure 2).

Five months later, the patient complained of deteriorated visual acuity to the level of light sensation. The functions of CN III and V2 were similar to that after 1^st ^CCRT. Follow-up MRI revealed the tumor recurred at left CS. CCRT for the recurrence was implemented and the disease has been in a stable condition. However, the vision in his left eye was completely loss after the 2^nd ^CCRT.

## Discussion

According to the World Health Organization histological classification published in 2005, malignant changes in the PA include three different types: CXPA, carcinosarcoma, and metastasizing PA [3,4]. CXPA is the most common malignant change and accounts for 11.7% of salivary malignancies [2-4]. Most of the malignant changes occur in the parotid gland and are extremely rare in minor salivary gland [6]. There are only less than 7% of CXPA found in the minor salivary glands [3]. The clinical presentation of CXPA may be similar to that of PAs and the most common symptom was an asymptomatic mass and sometimes there is a longstanding history of PA [1,7]. About 30% of patients present symptoms and signs including pain, facial nerve palsy, enlarged lymph nodes, skin ulceration and dysphasia [1,2]. CXPA usually occurs in the 6^th^~8^th ^decades of life [7]. The time from onset of symptoms until diagnosis varied from 1 month to 52 years [2].

The imaging findings of CXPA are usually nonspecific and difficultly identified from those of other benign and malignant salivary gland tumors [4]. PA shows a variety of signal intensities due to the cytomorphologic and architectural variabilities and is hard to be differentiated from low-grade malignant tumors in the absence of an irregular margin or infiltration into the surrounding tissue [4]. The diagnosis of CXPA with multiple invasions was eventually found by the pathological examinations. The local invasion from left maxilla to the CS was also demonstrated. Through the literature, abducens nerve palsy caused by the metastatic tumor in the CS had been reported [3,7,8] but there is no CXPA with CS invasion documented before. The tumor in the soft palate is readily to spread by way of greater palatine nerve (within the pharyngomaxillary fissure) toward the pterygopalatine fossa (PPF). Once the tumor lodges in the PPF, there are 4 possible pathways via which the tumor can choose to "travel": (1) anteriosuperiorly to the inferior orbital fissure to extraconal space; (2) posterosuperiorly to the foramen rotundum (V2) to the CS; (3) laterally to the masticator space, then the trigeminal nerve gets invaded and exploited as a route to the foramen ovale, dural layer and CS; (4) medially via the sphenopalatine foramen to the nasal cavity. Once the CS is invaded, the cranial nerves that pass through the leaves of the CS or within the sinus itself are affected, including cranial nerves III, IV, and VI as well as the V1 and V2 branches of the trigeminal nerve. Patients with CS invasion can present with headache, chemosis, proptosis, exophthalmos, and/or disturbed vision. The tumor in our patient was small in soft palate, but evident local invasion and perineural spread into the CS was seen on MRI. The perineural tumor spread may be asymptomatic; therefore, imaging studies play a critical role in the evaluation. Imaging findings of perineural spread of tumor include thickening and enhancement in the involved nerve, a widened foramen at the skull base, and obliteration of fat around the nerve as it exits the skull base. A combination of T1-weighted images, with and without contrast and fat saturation, best displays perineural tumor spread [9-11]. In our patient, the initial sign of CS invasion was cheek numbness. It is due to the large sizes of maxillary and mandibular divisions of the trigeminal nerve as they pass through the pterygopalatine fossa and are vulnerable to invasion [9-12].

There are controversies on the treatment of CXPA in salivary gland. The incidence of positive margins, perineural invasion, facial nerve involvement, and lymph node metastasis in CXPA is higher than other malignancies of the parotid gland. Post-operative adjuvant radiation could effectively eradicate residual deposits of microscopic disease [13]. CXPA also shows the characteristics of frequent recurrence and metastasis [7]. Metastatic lesions most frequently occur in regional lymph nodes, and some of them are seen in lung and bone [14]. Currently, surgery and post-operative adjuvant radiation therapy is accepted to improve local tumor control and a better tumor control is further associated with increased survival [8,13]. The 5-year survival rate of CXPA ranges from approximately 25% to 65% [14]. In our patient, complete excision of the tumor in CS was infeasible and it was further controlled by post-operative CCRT. Although the tumor recurred near CS later, re-irradiation is applicable and could achieve tolerable local tumor control.

## Conclusion

CXPA in the minor salivary gland with direct CS invasion was very rarely met. Our case is exceptional because the size of tumor in soft palate was small and not growing into the oral cavity. Instead, it spreads extensively through PPF and directly into the CS. It illustrates an atypical clinical presentation of the CXPA and the difficulty in its management.

## Competing interests

The authors declare that they have no competing interests.

## Authors' contributions

CHH and HSF conceived the idea for the manuscript, conducted a literature search, and drafted the manuscript. HSF performed surgery, obtained images and critically revised the manuscript. LLY provided and reviewed pathological images. CSC obtained radiological images used in the manuscript. CIH and LCT critically revised the manuscript. All authors read and approved the final manuscript.

## Consent

Written informed consent was obtained from the patient for publication of this case report and accompanying images. A copy of the written consent is available for review by the Editor-in-Chief of this journal.
